# Genome‐wide association mapping identifies novel loci underlying fire blight resistance in apple

**DOI:** 10.1002/tpg2.20087

**Published:** 2021-03-01

**Authors:** Ranjita Thapa, Jugpreet Singh, Benjamin Gutierrez, Jie Arro, Awais Khan

**Affiliations:** ^1^ Plant Pathology and Plant‐Microbe Biology Section Cornell University Geneva NY 14456 USA; ^2^ USDA–ARS Plant Genetic Resources Unit New York State Agricultural Experiment Station 630 West North Street Geneva NY 14456 USA

## Abstract

Fire blight, caused by epiphytotic gram‐negative bacteria *Erwinia amylovora*, is the most destructive bacterial disease of apple (*Malus* spp.). Genetic mechanisms of fire blight resistance have mainly been studied using traditional biparental quantitative trait loci (QTL) mapping approaches. Here, we use large‐scale historic shoot and blossom fire blight data collected over multiple years and genotyping‐by‐sequencing (GBS) markers to identify significant marker–trait associations in a diverse set of 566 apple [*Malus domestica* (Suckow) Borkh.] accessions. There was large variation in fire blight resistance and susceptibility in these accessions. We identified 23 and 38 QTL significantly (*p* < .001) associated with shoot and blossom blight resistance, respectively. The QTL are distributed across all 17 chromosomes of apple. Four shoot blight and 19 blossom blight QTL identified in this study colocalized with previously identified QTL associated with resistance to fire blight or apple scab. Using transcriptomics data of two apple cultivars with contrasting fire blight responses, we also identified candidate genes for fire blight resistance that are differentially expressed between resistant and susceptible cultivars and located within QTL intervals for fire blight resistance. However, further experiments are needed to confirm and validate these marker–trait associations and develop diagnostic markers before use in marker‐assisted breeding to develop apple cultivars with decreased fire blight susceptibility.

## Core Ideas

- Wide range of susceptibility responses to fire blight exists in *Malus domestica* germplasm
- GWAS using historical fire blight data of 566 *Malus domestica* accessions and GBS markers
- 23 and 38 minor‐moderate effect QTL identified for shoot and blossom fire blight respectively
- 23 fire blight QTL colocalized with previously identified disease resistance QTL in apple
- Transcriptomics data identified candidate genes in the QTL regions for fire blight resistance

**Abbreviations**
DEG: differentially expressed gene
FDR: false discovery rate
GBS: genotyping‐by‐sequencing
GO: gene ontology
GWAS: genome‐wide association studies
HAI: hours after infection
LD: linkage disequilibrium
LG: linkage group
MLM: mixed linear model
NB‐ARC: nucleotide‐binding adaptor shared by APAF‐1, R proteins, and CED‐4
QTL: quantitative trait loci
SNP: single nucleotide polymorphism.

## 1 INTRODUCTION

Fire blight, caused by the epiphytotic gram‐negative bacteria *Erwinia amylovora*, has remained a destructive disease of apple [*Malus domestica* (Suckow) Borkh.] for over 200 yr (Baker, 1971). Fire blight epidemics have caused significant annual economic losses of US$1.6 million in southern Germany (Scheer, 2009), US$9 million in Switzerland (Hasler et al., 2001), and US$100 million in the United States in pome fruit production (Norelli, Jones, & Aldwinckle, 2003). Fire blight bacteria enter through natural openings or wounds in host plants (Peil et al., 2009) and infect blossoms, leaves, vegetative shoot, fruits, woody tissues, and rootstock crown and suckers, which causes blossom, shoot, and rootstock blights, respectively (Norelli et al., 2003). Typical symptoms of blossom blight include water‐soaked and dull, grayish‐green appearance of floral receptacle and peduncle, which later shrivel and turn black. Symptoms of shoot blight include wilting shoot tips, production of bacterial ooze from the infected shoot, and eventually numerous shoots with fire blight infection give a burnt and blighted appearance to an infected tree. Blossom blight mainly reduces fruit yield in the current season, while shoot blight affects the fruit yield of following season by destroying the annual wood‐bearing fruit spurs. The subsequent progression of bacteria into the tree trunk can eventually destroy the whole tree (Peil et al., 2009). During the bloom period, hail, rain, and pollinating insects can spread the disease from infected blossoms to healthy trees in the orchard. In this scenario, developing blossom, shoot, and rootstock blight‐resistant cultivars is a promising way to sustainably manage fire blight in apple (Norelli et al., 2003).

Previous studies have identified major‐, moderate‐, and minor‐effect fire blight resistances from wild *Malus* species and domesticated apple (Calenge et al., 2005; Durel, Denancé, & Brisset, 2009; Khan, Duffy, Gessler, & Patocchi, 2006; Korban, Ries, Klopmeyer, Morrisey, & Hattermann, 1988; Le Roux et al., 2010; Peil et al., 2009). The major resistances were mainly identified in wild species, including Siberian crabapple (*M. ×robusta* 5), Japanese crabapple (*M. floribunda* Siebold ex Van Houtte sel. 821), *M. ×arnoldiana* (Rehder) Sarg. ex Rehder, and ornamental apple cultivar Evereste, each of which bear poor fruit quality traits (Kellerhals, Spuhler, Duffy, Patocchi, & Frey, 2007; Kostick, Norelli, & Evans, 2019). Therefore, breeding apple cultivars with both desirable market quality fruit and fire blight resistance is very challenging because of linkage drag, self‐incompatibility, and long generation time in apple (Aldwinckle, Gustafson, & Forsline, 1998; Peil et al., 2009; Van Der Zwet, 1979). Apple cultivars and varieties have also shown a wide range of fire blight susceptibility responses, providing a faster alternative to breed cultivars with decreased susceptibility or moderately improved fire blight resistance (Aldwinckle, Way, Livermore, Preczewski, & Beer, 1976; Kellerhals et al., 2007; Le Lézec, Babin, & Lecomte, 1990; Thibault & Le Lézec, 1990; Zeller, 1990). Cultivars such as Delicious, Stayman, Winesap, Florina, Liberty, and MacFree showed moderate to high fire blight resistance among evaluated apple (Aldwinckle et al., 1976, 1998). The varying fire blight resistance in some of these cultivars was also documented in successive studies (Brown, 2012; Mohan, Fallahi, & Bijman, 2001). Moderate to high levels of fire blight resistance has also been identified in cultivars Empire, Enterprise, Fireside, Frostbite, Liberty, Dolgo, Tsugaru, Wildung, and William's Pride (Kostick et al., 2019). The underlying genetic mechanism of fire blight resistance of some of these cultivars has been characterized (Peil, Emeriewen, Khan, Kostick, & Malnoy, 2020). However, the genetic basis of minor to moderate fire blight resistance of these cultivars is complex and not fully understood. Additionally, fire blight resistance in apple is of particular interest to breed fire blight resistant cultivars because of many desirable horticultural and fruit quality traits (Peil et al., 2020). The evaluation of historical blossom and shoot fire blight data for 694 and 2,362 *Malus* accessions, respectively, available in USDA–GRIN dataset, showed low to moderate susceptibility against fire blight in apple accessions (Khan & Chao, 2017). Approximately 21 and 60% apple accessions were characterized as resistant and highly susceptible, respectively, providing an opportunity to detect novel resistance alleles in apple (Khan & Chao, 2017).

However, only a few studies have characterized the genetic basis of these moderate and minor fire blight resistance in apple so far. A major quantitative trait loci (QTL) explaining 40% of the fire blight variance was identified in cultivar Fiesta (Calenge et al., 2005; Khan et al., 2006). The origin of this QTL on linkage group (LG) 7 (LG number corresponds to chromosome number) was traced back to cultivar Cox's Orange Pippin (Khan et al., 2007). Recently, a QTL in a similar position at LG07 was also reported in the fire blight resistant cultivar Enterprise (van de Weg et al., 2018). This QTL is considered most promising for fire blight resistance breeding because of its moderate and stable effect and apple background, but still, it needs fine mapping and in‐depth characterization to identify the underlying gene. Similarly, another strong QTL explaining 50–70% of fire blight variation was identified on LG12 in ornamental cultivar Evereste (Durel et al., 2009). Several other QTL identified from domesticated backgrounds had minor effects associated with fire blight infection responses (Calenge et al., 2005; Desnoues et al., 2018; Khan, Zhao, & Korban, 2013). The development of molecular markers linked to resistance QTL can increase the selection efficiency in breeding programs.

**Core Ideas**

- Wide range of susceptibility responses to fire blight exists in *Malus domestica* germplasm
- GWAS using historical fire blight data of 566 *Malus domestica* accessions and GBS markers
- 23 and 38 minor‐moderate effect QTL identified for shoot and blossom fire blight respectively
- 23 fire blight QTL colocalized with previously identified disease resistance QTL in apple
- Transcriptomics data identified candidate genes in the QTL regions for fire blight resistance

The fire blight QTL reported above were mainly identified through traditional biparental QTL mapping approaches (Khan & Korban, 2012; Malnoy et al., 2012; Peil et al., 2020). The QTL discovery in biparental crosses is restricted to the parental combinations used to create the mapping population. In contrast, genome‐wide association studies (GWAS) use historic recombination in large pools of genetically diverse accessions to evaluate greater diversity of alleles and haplotypes to provide finer QTL mapping resolution (Khan & Korban, 2012; Myles et al., 2009; Yu & Buckler, 2006; Zhu, Gore, Buckler, & Yu, 2008). The success of GWAS depends on the nature of diverse accessions in GWAS panel, reliable phenotypic data and dense molecular markers distributed across the genome. The use of GWAS is particularly challenging in apple for identifying marker–trait associations because of their high heterozygosity, rapid linkage disequilibrium (LD) decay, and recent genome duplication (Bianco et al., 2016; Velasco et al., 2010). A high‐quality reference genome and several high‐throughput genotyping platforms are already available for apple (Bianco et al., 2014; Bianco et al., 2016; Daccord et al., 2017; Velasco et al., 2010). The USDA–ARS Plant Genetic Resources Unit, Geneva, NY, holds ∼5,196 *Malus* accessions from 52 primary and secondary *Malus* species, representing wide diversity suitable for GWAS. This germplasm has been successfully used to identify QTL and candidate genes for fruit juiciness, fruit firmness, flesh mealiness, soluble solids content, and titratable acidity in apple fruit (Amyotte, Bowen, Banks, Rajcan, & Somers, 2017; Kumar et al., 2013; Kumar, Raulier, Chagné, & Whitworth, 2014; Migicovsky et al., 2016; Moriya et al., 2017). Similarly, use of historical disease data collected over many years from germplasm repositories for curation can be useful for genetic mapping and gene discovery (Mcclure et al., 2018) and can avoid the technical, labor, and space challenges associated with grafting, replications, management, maintenance, and controlled inoculations of large *Malus* germplasm for GWAS (Khan & Chao, 2017). Historical phenotypic datasets have been successfully used for GWAS in several crops, for example apple (Larsen et al., 2019; Migicovsky et al., 2016), barley (*Hordeum vulgare* L.) (Matthies, Malosetti, Röder, & Van Eeuwijk, 2014), grapevine (*Vitis vinifera* L.) (Migicovsky et al., 2017), and soybean [*Glycine max* (L.) Merr.] (Bandillo et al., 2015).

In this study, historical blossom and shoot fire blight data collected over 10 yr, available in USDA–GRIN global website, and genotyping‐by‐sequencing (GBS) data of 566 apple accessions were used to identify QTL linked with fire blight resistance using GWAS. We further identified potential candidate genes under QTL regions by using transcriptomics data from two apple cultivars with contrasting responses to fire blight infection.

## 2 MATERIALS AND METHODS

### 2.1 Blossom and shoot fire blight data collection, filtering, and analysis

The blossom and shoot fire blight data from 2,362 and 694 *Malus* accessions were downloaded from the USDA–GRIN global website (http://www.ars-grin.gov/gen/apple.html). These accessions represent more than 40 primary and secondary (inter‐species hybrids) *Malus* species planted at the USDA–ARS Plant Genetic Resources Unit, Geneva, NY. We have removed all accessions of wild *Malus* species from the dataset and only *M. domestica* accessions were kept for GWAS analysis in order to decrease the complexity of population genetic structure and avoid false positive marker–trait associations (Supplemental File S1).

The infection severity of shoot blight was recorded annually on each accession beginning in 1991 to 2000 (10 yr). The scale used to record both shoot and blossom infection severity data ranged from 1 to 5 (Table 1). At the end of these observations on the natural occurrence of fire blight in USDA apple germplasm, the highest rating score for shoot blight was assigned to each accession (Forsline & Aldwinckle, 2001). Blossom blight was recorded only once in 2000 following a severe blossom blight epidemic.

**TABLE 1:** Description of rating scores used to evaluate shoot and blossom fire blight infection severity. A rating score of 1 to 5 is given based on the level of resistance or susceptibility of the *Malus domestica* accessions to shoot and blossom fire blight infection (Forsline & Aldwinckle, 2001)

Rating score	Rating	Description of shoot blight	Percentage of blossom infection
			%
1	Very resistant	No occurrence	0
2	Moderately resistant	No fire blight lesions proceeded past 50% of current season growth on any shoots	1–5
3	Intermediate	Lesions would occasionally move to 2‐yr wood	5–15
4	Moderately susceptible	Heavy infection on many shoots in the tree together with an occasional lesion in major scaffolds	16–30
5	Extremely susceptible	Overall very heavy infection, often with lesions in trunks of trees requiring whole tree removal; in some cases causing tree death	31–100

We plotted the blossom and shoot fire blight infection scores according to the orchard layout in Microsoft excel version 16.16.25 (https://office.microsoft.com/excel) to visualize their distribution across all apple accessions. The accessions labeled as highly resistant (rating score = 1) for shoot blight were removed, as some of them could have escaped infection as a result of no disease inoculum (Khan & Chao, 2017). A final dataset comprised of 566 and 273 apple accessions, from 20 different countries, was used for GWAS analysis of shoot and blossom blight, respectively (Supplemental File S1).

The disease rating scores for all the accessions were used to perform correlation analysis between shoot and blossom blight and to plot frequency distribution graphs with ggplot package in R version 4.0.2 (Wickham, 2016). The correlation between ordinal–ordinal phenotypic traits of shoot and blossom blight were tested using Kendall's tau correlation test (Kendall, 1975).

### 2.2 GBS library construction, SNP calling, and SNP filtering

The GBS data used in this study is the same as used by Migicovsky et al. (2016). In brief, the GBS data was produced using the protocol described in Gardner et al. (2014), where GBS libraries were prepared using HindIII‐HF and Mspl (New England Biolabs, Ipswich, MA) by modifying original GBS protocol developed by Elshire et al. (2011) and Poland, Brown, Sorrells, and Jannink (2012). Afterward, clean GBS sequence reads were aligned against ‘Golden Delicious’ genome assembly v.2 (https://www.rosaceae.org/; Velasco et al., 2010) using bwa (Li & Durbin, 2009) and genome analysis toolkit, GATK (Mckenna et al., 2010), was used to call single nucleotide polymorphisms (SNPs).

We have used default parameters in Beagle version 5.1 (Browning & Browning, 2007) to impute the missing genotypic data and then to phase the GBS data. The SNPs with minor allele frequency of <.05 and missing data of >20% were excluded and only biallelic SNPs were retained using bcftools 1.10 (Danecek et al., 2014). The resulting genotyping data contained 566 accessions and 7,176 SNPs for shoot blight, and 273 accessions and 6,923 SNPs for the blossom blight.

### 2.3 Population genetic structure

The genetic structure and variance present in the GWAS panel comprised of apple accessions was investigated using principal component (PC) analysis with the genome‐wide SNP data to account for false positive marker–trait associations. Principal components were visualized using a PC analysis plot. The SNP dataset was further used to investigate the subpopulation structure with Bayesian clustering algorithm in the fastStructure program (Raj, Stephens, & Pritchard, 2014). The command ‘structure.py’ was run at different levels of *K* from 1 to 10 and command ‘choose K.py’ was used to identify the model complexity that maximized the marginal likelihood. The coefficient of ancestry (*Q*) threshold was defined at 70% to infer the individual ancestry from one particular subpopulation. The accessions lacking clear assignment to only one group were determined as admixtures. The population structure was visualized by plotting 3D scatterplot of top three PCs.

### 2.4 Model comparison and association mapping

The mixed linear model (MLM) was found to be the most promising model for GWAS using binary, categorical, or continuous traits in an association panel exhibiting population structure (M. Wang et al., 2012). The MLM model has been successfully used to identify candidate genes associated with categorical or binary traits in rice (*Oryza sativa* L.) (Huang et al., 2010), corn (*Zea mays* L.) (Romay et al., 2013), barley (M. Wang et al., 2012), and soybean (Rincker, Lipka, & Diers, 2016; Sonah, O'donoughue, Cober, Rajcan, & Belzile, 2015; Wen, Boyse, Song, Cregan, & Wang, 2015). We used unified MLM in GAPIT (Genome Association and Prediction Integrated Tool) package (Lipka et al., 2012). Both population structure (**Q** matrix) and kinship relationship matrix (**K** matrix) were used in the model to control spurious associations. The structure data (**Q**) was obtained from the fastStructure (Raj et al., 2014) as described above, and the relationship matrix (**K** matrix or kinship) was obtained from TASSEL 5.0 (Bradbury et al., 2007). The MLM model used was as follows: **Y**  = β**X** + *γ* **P** + **Z** *u* + ε, where **Y** is the vector of phenotype data, **X** is the vector of genotype data, β represents the SNP effects, **P** is a vector of the **Q** matrix resolving population structure, *γ* is the effect of population structure, *u* refers to the random effect from kinship, **Z** is a kinship matrix, and ε corresponds to random error.

The significance threshold for marker–trait association was set at *p* < 1.1 × 10^−3^. However, more stringent thresholds of false discovery rate (FDR) adjusted *p* value were also calculated separately for each trait. Both percentage variance explained by all significant SNPs and Manhattan plot for SNP distribution for each trait were obtained from GAPIT output. The percentage variance explained by each significant SNP was calculated as the difference between *R* ^2^ value of the model with and without SNP. The phenotypic effect of all the significant alleles (FDR < .05) were evaluated across different accessions using JMP Pro 14 (JMP, version 14, SAS Institute Inc., Cary, NC, USA 1989–2019).

### 2.5 Linkage disequilibrium decay

Linkage disequilibrium was calculated within 1000 kilobase (kb) pairs genomic region of the significant SNPs associated with shoot and blossom fire blight. The squared correlation of allele frequency (*r* ^2^) within 1000 kb pairs of significant SNPs was estimated using Tassel v 5.0 (Bradbury et al., 2007). The rate of LD decay was visualized by plotting squared correlation of allele frequency (*r* ^2^) and map distance (bp) using a LOESS smoothing line of ggplot2 package in R (Wickham, 2016). The LD decay plot was used to delimit the genomic regions for candidate gene search.

### 2.6 Candidate gene search in the QTL regions

All the potential candidate genes and the associated markers identified from previous studies (250 kb upstream and 250 kb downstream) of the significant loci were selected using GBrowse tool in Rosacea.org (Jung et al., 2014) and Hidras (Gianfranceschi & Soglio, 2003) for *M. domestica* reference genome assembly v.2. The sequence data of the identified markers and QTL were retrieved from Hidras (Gianfranceschi & Soglio, 2003) and aligned with reference genome with genome database for Rosaceae nucleotide BLAST tool (Jung et al., 2014) to determine the position of the markers and QTL in the genome. The positions of the significant SNPs identified in our study were compared with the position of the markers and the QTL reported in previous studies. Gene annotation information from apple was used to assign the functions of identified potential candidate genes (Velasco et al., 2010).

### 2.7 Differential gene expression in QTL regions

The transcriptomics data of two apple cultivars, Empire and Gala, which were previously found to have contrasting responses to fire blight infection (Silva, Singh, Bednarek, Fei, & Khan, 2019), were used to find differentially expressed genes (DEGs) and potential candidate genes in the LD region of significant SNPs. Empire was found to be significantly more tolerant than Gala based on the percentage lesion length and area under disease progress curve calculated 4, 6, 9, 12, and 15 d after inoculation with *E. amylovora* (Silva et al., 2019). The fragments per kilobase of transcript per million mapped reads counts of both control and treated samples of tolerant (Empire) and susceptible (Gala) cultivars at 24, 48, and 72 h after fire blight infection were used to detect DEGs with DESeq2 v1.16.1 (Love, Huber, & Anders, 2014). The list of candidate genes detected at 24, 48, and 72 h after inoculation (HAI) were provided to agriGO analysis tool kit (Tian et al., 2017) for gene ontology (GO) term analysis. A FDR value <.05 from Fisher statistical method was used to declare the significant enrichments. The significant (*p* value < .05) DEGs identified at each time points were compared pairwise to find if any DEGs were common across different time points. The DEGs discovered in the vicinity of significant SNPs, that is, within ±250 kb genomic region were extracted and compared with the gene annotation file (Velasco et al., 2010) to identify the functions of these potential candidate genes.

## 3 RESULTS

### 3.1 Variation in blossom and shoot fire blight in apple

**FIGURE 1:**
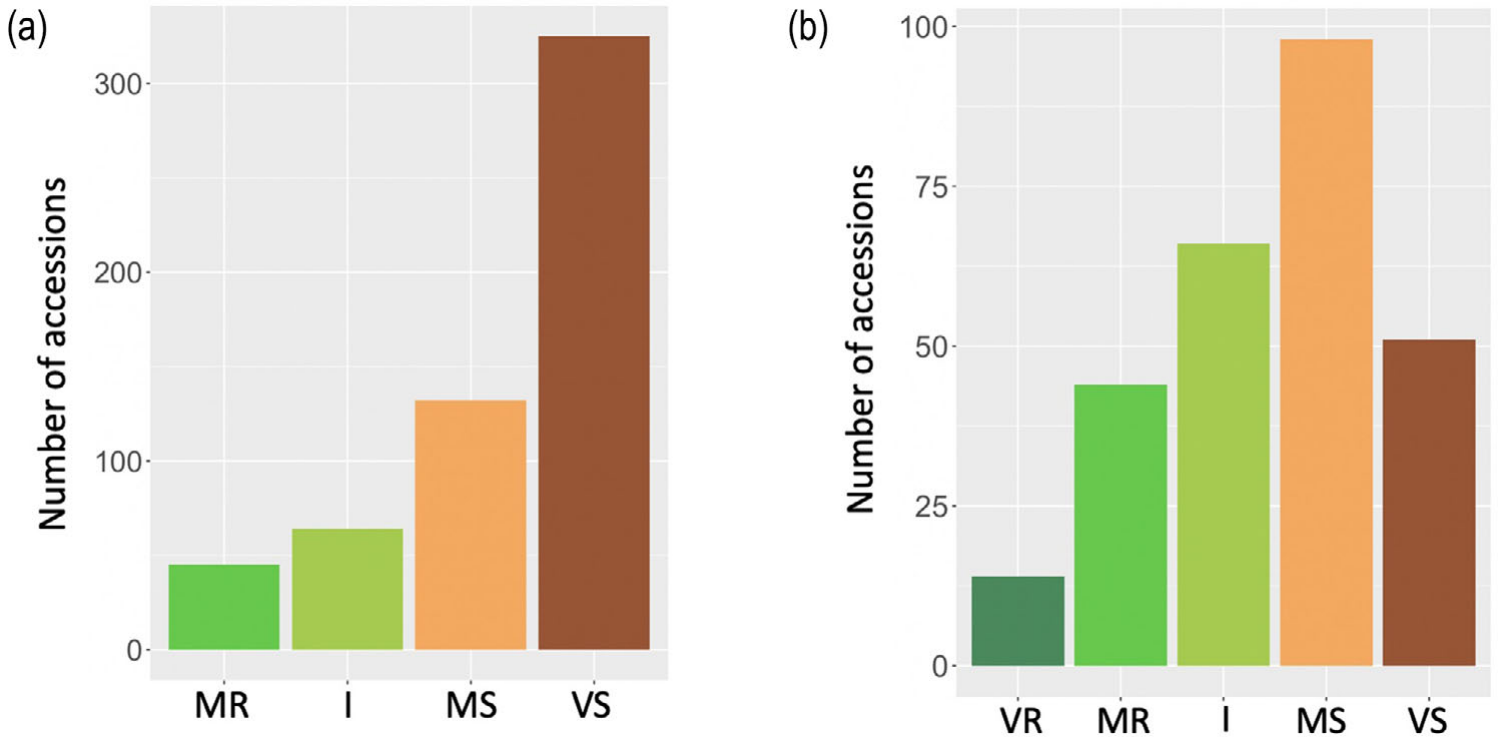
Distribution of fire blight resistance/susceptibility data of the 566 *Malus domestica* accessions used in genome‐wide association studies (GWAS); (a) shoot blight and (b) blossom blight. The *y* axis represents total number of accessions in each category of fire blight resistance or susceptibility and the abbreviations on *x* axis (VR, very resistant; MR, moderately resistant; I, intermediate; MS, moderately susceptible; VS, very susceptible) indicate disease rating scores. This historical fire blight data was downloaded from USDA–GRIN global website

A wide range of variation was observed in the GWAS panel for both blossom blight and shoot blight (Figure 1). Out of 566 accessions, a total of 45 (7.9%), 64 (11.30%), 132 (23.32%), and 325 (57.42%) accessions had a disease rating score of 2, 3, 4, and 5, respectively, for shoot blight infection. Similarly, 14 (5.12%), 44 (16.11%), 66 (24.17%), 98 (35.89%), and 51 (18.68%) accessions had a disease rating of 1, 2, 3, 4, and 5 for blossom blight, respectively. The correlation value estimate (*R* ^2^) between shoot blight and blossom blight was .22.

A total of 260 accessions were common between the GWAS panels for blossom blight and shoot blight. A comparative analysis of these common 260 accessions showed that 7, 10, 24, and 39 accessions had the same disease rating score of 2, 3, 4, or 5 for blossom and shoot blight datasets, respectively (Supplemental Table S1). For example, the cultivars Liberty and Fruitland Delicious exhibited moderate resistance (rating scale of 2) in both shoot and blossom blight infection datasets. Similarly, cultivars Cortland, Turley, Sunset, and Noret had intermediate resistance (rating scale of 3); cultivars Noran, Antonovka Shafron, Granny Smith, and Webster were moderately susceptible (rating scale of 4); and cultivars Ellison's Orange, Novinka, Early McIntosh, and Kimball McIntosh (2‐4‐4‐4) were extremely susceptible (rating scale of 5) to both shoot blight and blossom blight infections.

### 3.2 Population structure in the apple germplasm

Based on model complexity and model component analysis from Bayesian clustering analysis, the optimal number of predicted subpopulations were *K* = 10 and *K* = 5 for shoot and blossom blight GWAS panels, respectively. A PC analysis did not show strong population structure in either GWAS panel as depicted by the variance explained by each PC (Supplemental Figure S1). The amount of variance explained by PC1 and PC2 in the shoot blight GWAS panel were ∼3.71 and ∼2.52%, respectively. Similarly, the amount of variance explained by PC1 and PC2 in GWAS panel for blossom blight infection were ∼4.21 and ∼3.16%, respectively. A close observation of the population structure did not show any significant clustering of the accessions based on their place of origin. The LD decay value in the nearby genomic region of the significant SNPs (±1000 kb) associated with shoot blight and blossom blight ranged from 250 to 500 kb (Supplemental Figures S2 and S3). A squared correlation of allele frequency (*r* ^2^) at .001 was used to determine LD decay threshold value.

### 3.3 QTL for blossom and shoot fire blight

**FIGURE 2:**
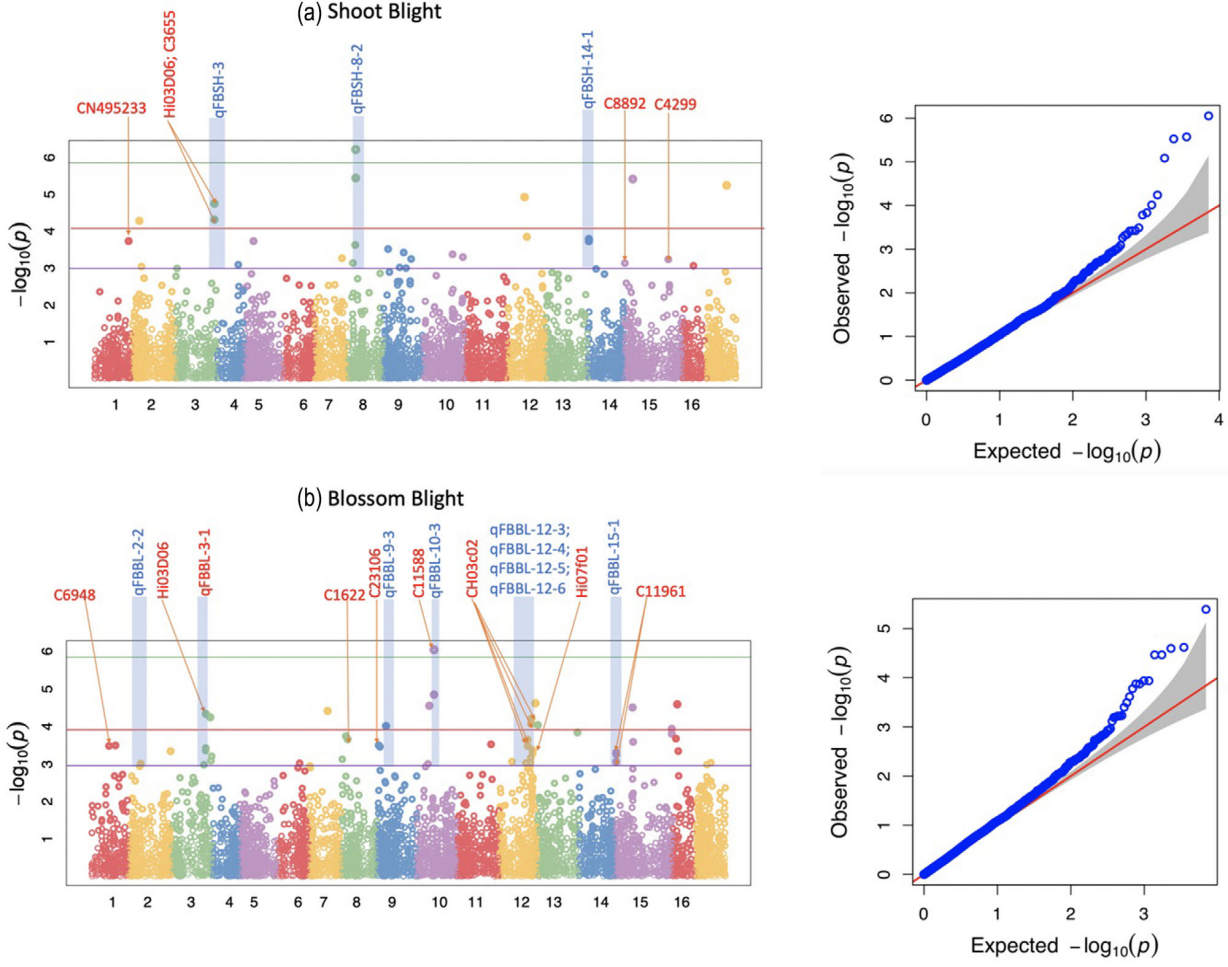
Manhattan plots show the level of significance for single nucleotide polymorphisms (SNPs) correlated with (a) shoot fire blight and (b) blossom fire blight in *Malus domestica* from genome‐wide association studies along with the corresponding Q‐Q plots on the right. Blue, red, and green horizontal lines indicate the threshold value of *p* < .001, false discovery rate corrected *P* value, and Bonferroni corrected *P* value, respectively. Chromosomal locations of reported SNP markers are shown with arrows. Blue transparent bars show quantitative trait loci identified in this study

We used a unified MLM model to conduct GWAS for the identification of significant marker–trait associations for blossom and shoot fire blight resistance in apple. A total of 49 and 27 significant (*p* < .001) markers were detected for blossom blight and shoot blight, respectively (Supplemental File S2). The amount of phenotypic variance explained by significant markers (*R* ^2^) ranged from 0.95 to 4.10% for shoot blight and 3.53 to 9.07% for blossom blight. There was a significant difference of causative alleles (FDR < .05) in the fire blight susceptibility and resistance of different accessions (Tables 2 and 3). Together, the significant markers for blossom and shoot fire blight were distributed across all 17 chromosomes of the apple genome (Figure 2), and no common QTL were identified between blossom and shoot blight traits. However, a significant QTL (qFBBL‐3‐2) for blossom blight on LG03 harboring a SNP GBS_35513256, and another significant shoot blight QTL (qFBSH‐3‐1) located on LG03 harboring two significant SNPs, GBS_35346100 and GBS_35346084, were located on the same 250‐kb window of LD block (Supplemental Figures S2 and S3).

**TABLE 2:** Significant (false discovery rate [FDR] < .05) single nucleotide polymorphisms (SNPs) associated with shoot fire blight resistance in *Malus domestica* identified using genome‐wide association studies

SNP^a^	Allele	+ +^b^	+ – ^b^	– – ^b^	QTL^c^	Chr^d^	*P* value	*R* ^2^	FDR	Effect	Distance	Colocalized marker	Reference
											kb		
Chr2_6989972	C/T	435(4.42)a	20(3)b	111(4.07)c	qFBSH‐2‐1	2	5.27 × 10^−5^	2.5	.047	−0.476	–	–	–
Chr3_35346084	A/G	435(4.37)a	43(4.04)b	88(4.09)b	qFBSH‐3‐1	3	4.89 × 10^−5^	2.6	.047	−0.271	199.48; 167.01; 22.40	*C3655*; *Hi03D06*; *MS14h03*	A. Wang et al., 2012; Emeriewen et al., 2014; Calenge et al., 2005
Chr3_35346100	A/G	439(4.36)a	41(3.88)b	86(4.18)ab	qFBSH‐3‐1	3	1.79 × 10^−5^	2.9	.021	−0.290	199.5; 167; 22.42	*C3655*; *Hi03D06*; *MS14h03*	A. Wang et al., 2012; Wöhner et al., 2014; Calenge et al., 2005
Chr8_7621442	A/T	444(4.37)a	15(3.6)b	107(4.1)b	qFBSH‐8‐2	8	6.16 × 10^−7^	4.1	.004	−0.440	–	–	–
Chr8_7621452	A/T	439(4.37)a	17(3.64)b	110(4.12)b	qFBSH‐8‐2	8	3.66 × 10^−6^	3.6	.009	−0.395	–	–	–
Chr12_15731421	C/A	27(3.85)b	439(4.42)a	100(3.92)b	qFBSH‐12‐1	12	1.20 × 10^−5^	2.6	.017	0.347	–	–	–
Chr15_8393790	G/A	497(4.36)a	69(3.85)b	–	qFBSH‐15‐2	15	3.89 × 10^−6^	3.4	.009	−0.608	203.68	*C8892*	Wang et al., 2012
Chr17_17949084	G/A	22(3.68)b	489(4.37)a	55(3.96)b	qFBSH‐17‐1	17	5.78 × 10^−6^	3.5	.010	0.404	–	–	–

^a^ The SNP name represents chromosome number and its corresponding physical position (cM) on it.

^b^ +, first allele; −, second allele. Number outside the bracket represents counts of accessions per genotypic category, number inside the bracket represents mean phenotypic rating. Lowercase letters represent statistical significance difference among phenotypes (*p* < .005) for each genotype category.

^c^ QTL, quantitative trait loci.

^d^ Chr, chromosome

**TABLE 3:** Significant (false discover rate [FDR] < .05) single nucleotide polymorphisms (SNPs) associated with blossom fire blight in *Malus domestica* identified using genome‐wide association studies

SNP^a^	Allele	+ +^b^	+ –^b^ ^b^	– – ^b^	QTL^c^	Chr^d^	*P* value	*R* ^2^	FDR	Effect	Distance	Colocalized marker	Reference
											kb		
Chr3_31530291	T/C	6(2.33)b	244(3.55)a	23(2.89)b	qFBBL‐3‐1	3	4.68 × 10^−5^	5.6	.0405	0.699	353.1	*MS14h03*	Calenge et al., 2005
Chr3_35513256	A/T	243(3.58)a	7(2.71)b	23(2.52)b	qFBBL‐3‐2	3	5.74 × 10^−5^	6.2	.043	−0.68	366.65; 156; 12.84	*C3655*; *Hi03D06*; *MS14h03*	Wang et al., 2012; Wöhner et al., 2014; Calenge et al., 2005
Chr7_16957826	C/T	235(3.58)a	2(1.5)b	36(2.83)b	qFBBL‐7‐1	7	3.89 × 10^−5^	6.5	.0384	−0.76	346.14	*CH04e05*	Calenge et al., 2005; Khan et al., 2006
Chr9_8096777	A/T	232(3.35)b	2(5)a	39(4.07)a	qFBBL‐9‐3	9	9.84 × 10^−5^	5.7	.0487	0.701	–	–	–
Chr9_8096786	A/C	232(3.35)b	2(5)a	39(4.07)a	qFBBL‐9‐3	9	9.84 × 10^−5^	5.7	.0487	0.701	–	–	–
Chr10_11491311	C/G	233(3.59)a	5(2.8)ab	35(2.74)b	qFBBL‐10‐2	10	2.83 × 10^−5^	6.7	.0364	−0.69	–	–	–
Chr10_15747585	G/A	10(2.1)a	237(3.57)b	26(3.03)c	qFBBL‐10‐3	10	1.43 × 10^−5^	7.5	.0364	0.652	–	–	–
Chr10_15747588	G/A	7(1.86)a	239(3.59)b	27(2.85)c	qFBBL‐10‐3	10	9.17 × 10^−7^	9.1	.0064	0.803	–	–	–
Chr12_29065946	G/A	15(2.73)b	217(3.59)a	41(3.04)b	qFBBL‐12‐6	12	8.46 × 10^−5^	5.6	.0487	0.489	808.01	*CH03c02*	Wöhner et al., 2014
Chr12_29298043	A/G	228(3.57)a	6(2)b	39(3.07)c	qFBBL‐12‐6	12	6.20 × 10^−5^	6.1	.043	−0.62	575.92	*CH03c02*	Wöhner et al., 2014
Chr12_32938449	T/C	4(2)b	225(3.57)a	44(3.07)b	qFBBL‐12‐8	12	2.43 × 10^−5^	5.8	.0364	0.686	–	–	–
Chr13_368459	G/A	7(2.14)b	248(3.54)a	18(2.89)b	qFBBL‐13‐1	13	9.24 × 10^−5^	5.3	.0487	0.68	–	–	–
Chr15_16733197	A/C	217(3.36)b	19(4.58)a	37(3.54)b	qFBBL‐15‐2	15	3.16 × 10^−5^	6.5	.0364	0.496	–	–	–
Chr16_4492076	A/T	238(3.57)a	6(2.17)b	29(2.89)b	qFBBL‐16‐1	16	2.59 × 10^−5^	6.7	.0364	−0.72	–	–	–

^a^ The SNP name represents chromosome number and its corresponding physical position (cM) on it.

^b^ +, first allele; −, second allele. Number outside the bracket represents counts of accessions per genotypic category, number inside the bracket represents mean phenotypic rating. Lowercase letters represent statistical significance difference among phenotypes (*p* < .005) for each genotype category.

^c^ QTL, quantitative trait loci.

^d^ Chr, chromosome

For blossom blight, 29 out of 49 markers occupied a single genomic location, while 20 markers were present across nine distinct genomic sites over 14 chromosomes. After considering markers as linked or unique QTL when within 250‐kb windows, 38 independent QTL remained for blossom fire blight. Out of these 38 QTL, nine QTL (qFBBL‐2‐2, qFBBL‐3‐1, qFBBL‐9‐3, qFBBL‐10‐3, qFBBL‐12‐5, qFBBL‐12‐6, qFBBL‐12‐7, qFBBL‐15‐1, and qFBBL‐15‐4) had more than one significant SNP. A similar linkage consideration led to detection of 23 distinct QTL for shoot blight. Among 23 significant QTL, three QTL (qFBSH‐3‐1, qFBSH‐8‐2, and qFBSH‐14‐1) showed significant association with more than one SNP. The QTL for blossom blight were detected on 14 chromosomes—all except chromosomes 4, 5, and 14. The highest number of QTL for blossom blight were eight on chromosome 12 followed by chromosome 3 and 15 with four QTL each. The QTL for shoot blight were also distributed on 14 chromosomes—all except chromosomes 6, 11, and 13. The four largest effect QTL for shoot blight were on chromosome 9, and three QTL were identified on chromosome 15. By using stringent FDR < .05 criteria, the number of significant QTL was reduced to 14 and eight for blossom blight (Figure 2; Table 2) and shoot blight (Figure 2; Table 3), respectively.

### 3.4 Colocalization with previously detected QTL and candidate genes for fire blight

A total of 19 and four distinct QTL for blossom and shoot blight, respectively, were located in close vicinity of previously reported genes related to disease resistance in apple (Figure 2; Tables 2 and 3). The four blossom blight QTL were detected within 100 kb of a previously identified marker associated with fire blight. The QTL, qFBBL‐1‐1, was located 74.79 kb from marker *C6948*, previously reported to be linked with scab resistance (A. Wang et al., 2012). Similarly, QTL qFBBL‐3‐2 was located 12.84 kb from marker *MS14h03*, QTL qFBBL‐12‐3 and qFBBL‐12‐4 were located 54.39 and 54.42 kb from *CH03c02*, and QTL qFBBL‐3‐3 was located 122.83 kb from *MS14h03*; all of which have been linked to fire blight resistance in apple (Calenge et al., 2005; Wöhner et al., 2014).

Significant QTL, qFBBL‐8‐2, qFBBL‐9‐1, qFBBL‐9‐2, and qFBBL‐10‐1, were located 176.55, 208.83, 218.04, and 162.77 kb from the markers linked to scab resistance genes *C1622*, *C23106*, *C10534*, *C11588*, respectively (A. Wang et al., 2012). Similarly, qFBBL‐3‐1, qFBBL‐3‐4, qFBBL‐7‐1, qFBBL‐12‐1, qFBBL‐12‐2, qFBBL‐12‐7, and qFBBL‐12‐6 QTL were within 250 kb of previously detected fire blight linked markers (Table 3). A similar analysis also identified previously detected QTL in the vicinity of shoot blight QTL from this study. For instance, qFBSH‐3‐1 was 22.4 kb away from fire blight resistance linked marker *MS14h03* (Calenge et al., 2005). The same QTL was found to be located 199.48 kb from scab resistance marker *C3655* (A. Wang et al., 2012). Two other significant QTL, GBS15_1274294 and GBS15_8393790, were 110.63 and 203.68 kb from markers *C4299* and *C8892* for scab resistance in apple, respectively (A. Wang et al., 2012). One significant QTL (qFBSH‐12‐2) was 1214.56 kb away from a marker *CH01g12* related to fire blight resistance (Calenge et al., 2005).

### 3.5 Differentially expressed genes and pathways in response to fire blight inoculation

We further used gene expression information from the apple cultivars Empire and Gala, known to have distinct fire blight responses (Silva et al., 2019), to identify candidate genes within the shoot and blossom blight QTL regions. This analysis identified 45, 89, and 94 DEGs (*p* value < .05) at 24, 48, and 72 HAI within the 250‐kb upstream and downstream genomic region of identified significant SNP (Supplemental File S3; Supplemental Figure S4). At 24 HAI, the DEGs represented genes from the nucleotide‐binding adaptor shared by APAF‐1, R proteins, and CED‐4 (NB‐ARC) domain‐containing disease resistance protein; salt tolerance zinc finger; WRKY DNA‐binding protein 33; jasmonic acid carboxyl methyltransferase; 6‐phosphogluconate dehydrogenase family protein; and TRAF‐like family protein categories. Similarly, DNAJ heat shock N‐terminal domain‐containing protein; disease resistance protein (TIR‐NBS‐LRR class) family; serine carboxypeptidase S28 family protein; ethylene responsive element binding factor 4; NB‐ARC domain‐containing disease resistance protein; early‐responsive to dehydration stress protein (ERD4); and myb‐like transcription factor family protein were among the DEGs identified at 48 HAI. At 72 HAI, DEGs from disease resistance protein (TIR‐NBS‐LRR class) family; peroxidase superfamily protein; serine–threonine‐protein kinase WNK (With No Lysine)‐related, leucine‐rich receptor‐like protein kinase family protein; senescence‐associated gene 101; and growth‐regulating factor 3 were identified under the significant QTL peaks. Pairwise comparison of DEGs identified across three different time points revealed 12, 11, and 7 DEGs common at 24 and 48 HAI, 48 and 72 HAI, and 24 and 72 HAI (Supplemental Figure S4). Many of these DEGs represented the disease‐resistance families such as MD03G1280000 and MD03G1280200 (NB‐ARC domain‐containing disease resistance protein) on chromosome 3 and MD02G1109700 [disease resistance protein (TIR‐NBS‐LRR class) family] on chromosome 2.

**FIGURE 3:**
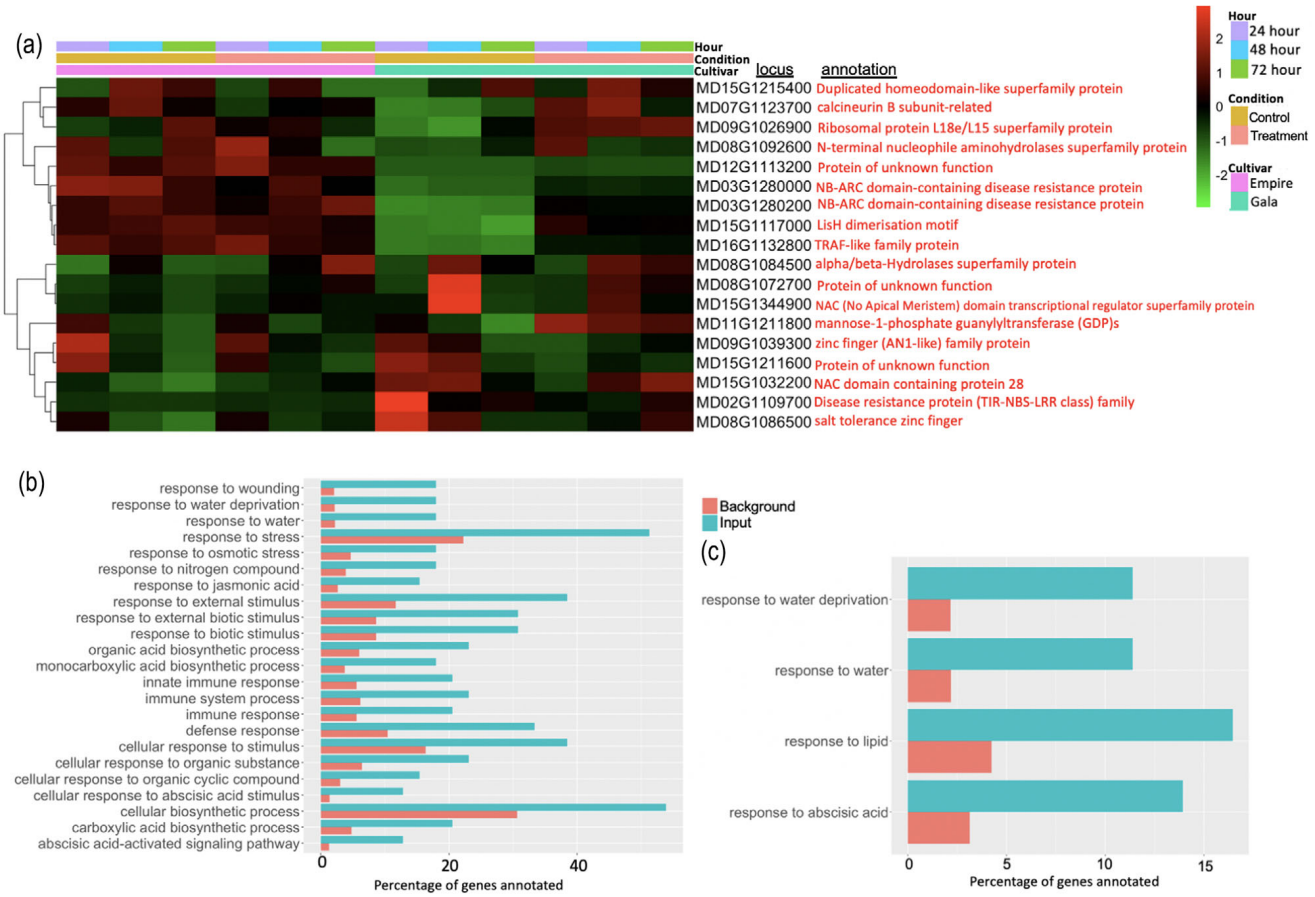
(a) Relative expression of 18 differentially expressed genes (DEGs) located within candidate loci discovered from the genome‐wide association studies in *Malus domestica*. These DEGs were identified as common in at least one pairwise comparison of DEGs at 24 h after inoculation (HAI), Hours after inoculation (48 HAI) and hours after inoculation (72 HAI) with *Erwinia amylovora* of shoots of tolerant cultivar Empire and susceptible cultivar Gala. (b) Gene ontology (GO) enrichment analysis of biological process of DEGs in two contrasting genotypes Empire (resistant) and Gala (susceptible) in response to fire blight inoculation on shoot at 24 HAI. (c) Gene ontology enrichment analysis of biological process of DEGs in two contrasting genotypes, Empire (resistant) and Gala (susceptible) in response to fire blight inoculation on shoot at 48 HAI. The number on the *x* axis represents percentage of annotated genes; the blue bar indicates enriched GO from input genes and red bar indicates enriched GO from genes of reference genome. Analysis was performed in agriGO with ‘Golden Delicious’ double haploid genome as reference. Only significantly enriched terms (false discovery rate < 5%) are shown

A GO analysis of DEGs, under significant QTL regions, identified 39 and five GO terms significantly (FDR < .05) enriched at 24 and 48 h, respectively (Supplemental File S3). Interestingly, many GO terms related to biological process potentially underlying disease resistance mechanism (Figure 3b,c) were significantly enriched at 24 and 48 HAI including GO:0009611 (response to wounding), GO:0009605 (response to external stimulus), GO:0043207 (response to external biotic stimulus), GO:0009607 (response to biotic stimulus), GO:0006952 (defense response), GO:0071215 (cellular response to abscisic acid stimulus), GO:0009738 (abscisic acid‐activated signaling pathway), GO:0002376 (immune system process), GO:0006955 (immune response), and GO:0045087 (innate immune response).

## 4 DISCUSSION

We observed ∼36 and ∼23% apple accessions in this historical dataset to be moderately susceptible to moderately resistant against blossom and shoot fire blight infections, respectively. These accessions included cultivars Cortland, Turley, Wellington, Fruitland Delicious, Tohuku, and Toto. The same accessions also showed a resistant response in 1972 and 1974 after severe fire blight infection at the Geneva experiment station (Aldwinckle et al., 1976). Fire blight severity ratings for cultivars Liberty (moderately resistant), Haralson (moderately susceptible), Cortland (intermediate), and Granny Smith (moderately susceptible) in this dataset showed similar responses as reported in a previous greenhouse experiment (Aldwinckle et al., 1998). Apple cultivars Delicious, Stayman, Winesap, Florina, Liberty, and Macfree showed moderately susceptible to moderately resistant responses against fire blight, similar to Mohan et al. (2001) and Brown, (2012). Recently, fire blight susceptibility evaluations of 94 apple cultivars in a controlled inoculation experiment in the orchard reported moderate to high levels of fire blight resistance in apple cultivars Empire, Enterprise, Fireside, Frostbite, Liberty, Dolgo, Tsugaru, Wildung, and William's Pride (Kostick et al., 2019). These greenhouse and field experiments suggest the presence of moderate to high levels of resistance in the apple germplasm. However, there are some inconsistencies in the fire blight response of different cultivars between different studies. For instance, Delicious has been reported as both susceptible and moderately resistant in different studies (Brown, 2012; Mohan et al., 2001). In addition, cultivar Monroe was highly susceptible in this historic fire blight dataset, but showed 23% infection under greenhouse evaluation in a previous study (Aldwinckle et al., 1998). The resistant cultivars, including Honey Ball, Kingston Black, Akane, Monroe, and Spigold from a previous study had susceptible disease scores in the historic fire blight dataset (Aldwinckle et al., 1976). There could be multiple reasons for some of these inconsistencies, including differences in phenotypic evaluation methods between different studies, co‐occurrence of multiple strains with different virulence mechanisms in the orchard, the nonrandom distribution of inoculum, and differences in environmental conditions can affect the infection severity and host response under greenhouse, field conditions, and across multiple studies (Khan & Chao, 2017; Peil et al., 2009). Particularly, the phenotypic responses controlled by minor and moderate genetic factors will have stronger impact from environment variables that can result in over‐ or underestimation of resistance ratings between different studies. Despite the possibility of false positive and false negatives, overall consistency in rating scores of accessions between different studies, provide opportunities to use the historical data for genetic characterization of fire blight resistance in apple. Historical data may represent more realistic host disease responses, closer to what can happen in the growers’ orchards.

The rating scores for both shoot blight and blossom blight for 80 accessions were the same with a weak correlation (*r* ^2^ = 0.22) between the two traits. Previously, both strong and weak correlations have been reported between floral and shoot inoculated fire blight responses (Peil et al., 2019; Thibault & Le Lézec, 1990). The strong correlation was explained by large genetic effect of the *Mr5* gene for fire blight resistance (Peil et al., 2019), while the weak correlation was reported to be caused by a strong environmental effect in the susceptible Gala cultivar (Thibault & Le Lézec, 1990). The weak correlation between blossom and shoot blight in the historic fire blight dataset can be attributed to strong environmental influence on minor effect QTL or absence of strong major effect QTL for these traits in the apple accessions. The identification of 23 and 38 unique minor‐effect QTL for shoot blight and blossom blight, respectively, can partially explain the different genetic mechanisms associated with these traits in apple background. Moreover, in comparison with previously reported fire blight QTL, 19 QTL associated with shoot blight and 19 QTL associated with blossom blight can be considered novel. A total of four shoot blight QTL and 10 blossom blight QTL showed colocalization with genes for fire blight or apple scab resistance. In particular, a previously known marker associated with fire blight, *MS14h03* (Calenge et al., 2005), was found to be <25 and <15 kb away from the significant QTL, qFBSH‐3‐1 associated with shoot blight and qFBBL‐3‐2 associated with blossom blight, respectively. In addition, one significant QTL associated with blossom blight, qFBBL‐3‐3, was found to be potentially colocalized with *MS14h03*, previously reported to be associated with fire blight resistance QTL identified in a population derived from a cross between cultivars Prima and Fiesta (Calenge et al., 2005). Two other QTL, qFBBL‐12‐3 and qFBBL‐12‐4, were found to be potentially colocalized with *CH03c02* on chromosome 12, previously reported to be associated with a fire blight resistance QTL identified in a population derived from a cross between cultivars Idared and *M. robusta* 5 (Wöhner et al., 2014). The colocalization of the significant QTL with previously known QTL highlights the stable effect of these QTL. A significant QTL explaining 15.3 to 17.9% variation in fire blight resistance identified on chromosome 10 of cultivar Florina (Le roux et al., 2010) was found to be clustered with scab resistance and powdery mildew resistance QTL (Calenge & Durel, 2006). The colocalization of scab resistance genes and fire blight resistance genes in our study could be due to clustering of the resistant genes. The plant resistant genes were reported to be clustered in the genome (Hulbert, Webb, Smith & Sun, 2001) and can serve as molecular markers for other genes controlling different traits.

The phenotypic variation explained by the identified QTL varied from 0.95 to 9.07%, indicating their minor to moderate contribution toward shoot and blossom fire blight resistance. Genetic background, population type and size, and genetic mapping approach can impact detection ability and over‐ or underestimation of allelic effects (Doebley, Stec, & Gustus, 1995; Tanksley & Hewitt, 1988). The genetic background of parents in biparental crosses in previous studies could have been the main reason to identify moderate to large effect QTL for fire blight (Emeriewen et al., 2014; Emeriewen et al., 2017; Khan et al., 2006; Peil et al., 2007). In more diverse GWAS panels, the effect attributable to rare alleles is hard to detect (Korte & Farlow, 2013). In contrast, diverse germplasm panels in GWAS with rapid LD decay, together with dense genome‐wide markers, increase the power and resolution of QTL identification (Khan & Korban, 2012). This might be the reason we identified a large number of minor‐effect QTL with narrow intervals in the current study.

The combined analysis of GWAS and transcriptomics identified enrichment for specific GO terms related to defense response, immune response, response to external stimuli, response to biotic stimuli, and response to abscisic acid under significant QTL peaks. Genes from disease‐related families including NB‐ARC domain‐containing disease resistance proteins, jasmonic acid carboxyl methyltransferase, zinc finger (AN1‐like), and WRKY DNA‐binding proteins were among the potential candidates under the most significant QTL peaks. These candidate genes have unique roles in different disease resistance mechanisms. For instance, the NB‐ARC domain‐containing genes have been characterized for a wide range of pathogen resistance in plants (Cai et al., 2008; Chandra et al., 2017; Wen et al., 2017). Ethylene and jasmonate were reported to play a defensive role against necrotrophic pathogens like *E. amylovora* (Glazebrook, 2005), and genes for these hormones have specific roles in responding to injury and biotic stress caused by insects and pathogens (Jin, Zheng, Tang, Rui, & Wang, 2009; Meng, Han, Wang, & Tian, 2009). A WRKY transcription factor was described to be involved in contributing to the rate of symptom development and resistance to the fire blight pathogen (Baldo et al., 2010). Similarly, peroxidase and a serine–threonine protein kinase were previously identified to be located near a fire blight resistance QTL on chromosome 3 (Norelli et al., 2008), and these genes also harbored significant QTL peaks in the current study. All together, these findings suggest a system‐wide interaction between genes from different families in response to fire blight infection. Moreover, some of these genes show differential expression at different time points after pathogen infection (Silva et al., 2019), hence suggesting the possible involvement of these QTL in pathogen perception and control.

In summary, large variation in susceptibility against fire blight in the historic dataset identified novel fire blight resistance QTL and potential candidate genes in apple. However, further experiments are needed to confirm and validate these QTL for marker‐assisted breeding in apple to develop cultivars with decreased fire blight susceptibility.

## AUTHOR CONTRIBUTIONS

Ranjita Thapa: Formal analysis; Writing‐original draft; Writing‐review & editing. Jugpreet Singh: Review & editing. Benjamin Gutierrez: Review & editing. Jie Arro: Review & editing. Awais Khan: Conceptualization; Funding acquisition; Project administration; Resources; Writing‐review & editing

## CONFLICTS OF INTEREST

The authors declare that they have no competing interests.

## Supporting information

Supplemental Figure S1. Genetic structure in *Malus domestica* accessions used for Genome‐wide Association Studies (GWAS) (a) PCA plot showing genetic structure of 566 *M. domestica* accessions used for shoot blight and (b) PCA plot showing genetic structure of 273 *M. domestica* accessions used for blossom blight, where different colored dots represent different subpopulations. The x, y, and z axes of PCA plot represent PC1, PC3, and PC2, respectively. The amount of variance explained by the corresponding PCs are given inside the corresponding parentheses.

Supplemental Figure S2. Detailed view of the peak regions associated with blossom fire blight on chromosomes 3 (a), 8 (b), 10 (c), 12 (d), and 16 (e) of *Malus domestica*. Pair‐wise linkage disequilibrium (LD) between single nucleotide polymorphism (SNP) markers is indicated as D' values: dark red indicates a value of 1 and white indicates 0. The red dots indicate significant SNPs above threshold of ‐log10(P) > 3. The x‐axis represents the position of SNPs in basepair (bp), y‐axis represents ‐log10(P) value.

Supplemental Figure S3. Detailed view of the peak regions associated with shoot fire blight on chromosomes 2 (a), 8 (b) and 12 (c) of *Malus domestica*. Pair‐wise linkage disequilibrium (LD) between single nucleotide polymorphism (SNP) markers is indicated as D' values: dark red indicates a value of 1 and white indicates 0. The red dots indicate significant SNPs above threshold of ‐log10(P) > 3. The x‐axis represents the position of SNPs in basepair (bp), y‐axis represents ‐log10(P) value.

Supplemental Figure S4. Venn diagram showing differentially expressed genes (DEGs) in the LD region of significant SNPs (*p* < 0.05) from genome wide association studies (GWAS). The DEGs were identified by comparing the normalized read counts between control and fire blight infected plants in the ‘Gala’ and ‘Empire’ apple cultivars using DESeq2 v1.16.1 (Love et al., 2014).

**Supplemental Table S1**. Accessions of *Malus domestica* with common rating score for both blossom fire blight and shoot fire blight. This historical fire blight data was downloaded from USDA–GRIN global website. Rating score for the level of resistance or susceptibility evaluation is based on Forsline and Aldwinckle, (2001).

**Supplemental File S1**. List of *Malus domestica* accessions and their respective PI number, plant identifier, genus name, species name, country of origin, population number (Identified from fastStructure) and rating score used for GWAS of blossom and shoot fire blight infection traits.

**Supplemental File S2**. List of significant SNPs associated with blossom and shoot fire blight identified at a threshold *P*‐value of < 0.001.

**Supplemental File S3**. Differentially expressed genes (DEGs) in the ‘Gala’ and ‘Empire’ apple cultivars at 24 hours after inoculation (HAI), 48 hours after inoculation (HAI) and 72 hours after inoculation (HAI) with *Erwinia amylovora* strain ‘Ea2002A’, common DEGs identified at 24, 48, and 72 HAI, and significantly enriched GO terms at 24 and 48 HAI.

**Supplemental File S4**. Shoot blight (FBSHNAT) and blossom (FBBLNAT) fire blight data for 273 and 566 *Malus domestica* accessions, respectively, with corresponding Genotyping‐by‐Sequencing (GBS) genotypic data used for GWAS. Phenotypic file also includes: PI (plant identifier) number, name, species name, and country of origin for the accessions.
