# Supplementary material for: Genome‐wide association mapping identifies novel loci underlying fire blight resistance in apple
Source: Plant Genome. 2021 Mar 1;14(2):e20087. doi: 10.1002/tpg2.20087 (PMC12807190; doi:10.1002/tpg2.20087)
Supplement: Supplementary file 1 [file TPG2-14-e20087-s004.docx]

**Supplemental Table 1.** Accessions of *Malus domestica* with common rating score for both blossom fire blight and shoot fire blight. This historical fire blight data was downloaded from USDA-GRIN global website. Rating score for the level of resistance or susceptibility evaluation is based on Forsline and Aldwinckle, (2001).

| Blossom fire blight and Shoot fire blight disease rating score | Common accessions |
| --- | --- |
| 2 | Alpha 68A, Wellington, M.7, Patul, Budagovsky 57-491, Liberty, Fruitland Delicious |
| 3 | Hunter Spy 2-4-4, Sublett, Delistein, Merton Beauty, Noret, Sunset, Tohoku 2, Cortland, Turley, Inducoa No. 1 |
| 4 | Dermen Paragon 6-3-3, Pomme Cloche, Surprise Reinette, Red Paducah, ShellinKout, Toyo, Goldgelb 55544, Haralson, Noran, Lindel, Narjadnoye Crysmskoye, Obilnoye, Joys, Schneiderapfel, Blenheim Orange, Pigeonnet, Antonovka Shafran, Uralian Winter Apple, 10/12/B, FO-59-4, Hordapfel, Granny Smith, Webster, Minnehaha |
| 5 | Autumn Arctic, King David, Cherry Pearmain, Mottais, BII, Merton Delight, Honey Ball, Kestrel, Vystavochnoye, Novinka, Ellison's Orange, Holaart Doux, Ellis Bitter, St. Edmund's Russet, Emneth Early, Brown's Apple, Sunburn, Court Royal, Merton Pearmain, Chiver's Delight, Kingston Black, Akane, Monroe, Sweet Caroline, M.10, M.13, Crittenden, Esopus Spitzenburg, Kingjon, Wemmershoek, Frumos de Voinesta, Kile, Potter, Nittany, Vista Bella, Spigold, Early McIntosh, Red Astrachan, Kimball McIntosh 2-4-4-4 |
